# C^2^Maps: a network pharmacology database with comprehensive disease-gene-drug connectivity relationships

**DOI:** 10.1186/1471-2164-13-S6-S17

**Published:** 2012-10-26

**Authors:** Hui Huang, Xiaogang Wu, Ragini Pandey, Jiao Li, Guoling Zhao, Sara Ibrahim, Jake Y Chen

**Affiliations:** 1 School of Informatics, Indiana University, Indianapolis, USA; 2 Indiana Center for Systems Biology and Personalized Medicine, Indianapolis, USA; 3 Department of Computer Science and Technology, Shandong College of Electronic Technology, Jinan, China; 4 Biology Department, School of Science, Indiana University, Indianapolis, USA

## Abstract

**Background:**

Network pharmacology has emerged as a new topic of study in recent years. It aims to study the myriad relationships among proteins, drugs, and disease phenotypes. The concept of molecular connectivity maps has been proposed to establish comprehensive knowledge links between molecules of interest in a given biological context. Molecular connectivity maps between drugs and genes/proteins in specific disease contexts can be particularly valuable, since the functional approach with these maps helps researchers gain global perspectives on both the therapeutic profiles and toxicological profiles of candidate drugs.

**Methods:**

To assess drug pharmacological effect, we assume that "ideal" drugs for a patient can treat or prevent the disease by modulating gene expression profiles of this patient to the similar level with those in healthy people. Starting from this hypothesis, we build comprehensive disease-gene-drug connectivity relationships with drug-protein directionality (inhibit/activate) information based on a computational connectivity maps (C^2^Maps) platform. An interactive interface for directionality annotation of drug-protein pairs with literature evidences from PubMed has been added to the new version of C^2^Maps. We also upload the curated directionality information of drug-protein pairs specific for three complex diseases - breast cancer, colorectal cancer and Alzheimer disease.

**Results:**

For relevant drug-protein pairs with directionality information, we use breast cancer as a case study to demonstrate the functionality of disease-specific searching. Based on the results obtained from searching, we perform pharmacological effect evaluation for two important breast cancer drugs on treating patients diagnosed with different breast cancer subtypes. The evaluation is performed on a well-studied breast cancer gene expression microarray dataset to portray how useful the updated C^2^Maps is in assessing drug efficacy and toxicity information.

**Conclusions:**

The C^2^Maps platform is an online bioinformatics resource that provides biologists with directional relationships between drugs and genes/proteins in specific disease contexts based on network mining, literature mining, and drug effect annotating. A new insight to assess overall drug efficacy and toxicity can be provided by using the C^2^Maps platform to identify disease relevant proteins and drugs. The case study on breast cancer correlates very well with the existing pharmacology of the two breast cancer drugs and highlights the significance of C^2^Maps database.

## Background

Screening millions of chemical compounds to identify "hit" compounds for specific disease gene/protein targets has been a mainstream paradigm for modern drug discovery [1]. While the conventional "One disease, One gene, and One drug" paradigm [2] works effectively for simple genetic disorders, it fails to produce effective drugs for complex diseases such as cancer [3]. In complex diseases, many genes may be contributing to the disease's phenotype; therefore, identifying a "magic bullet" drug compound can be quite elusive.

Polypharmacology, which focuses on multi-target drugs, has become a new paradigm in drug discovery. Polypharmacology drugs have conventionally been viewed to have undesirable 'promiscuity'. However, recent research studies show, in the case of both older psychiatric drugs and modern anticancer therapies, that this promiscuity is intrinsic to the drug's therapeutic efficacy [4]. Although there are over 40 drug-target (protein-compound interaction) databases according to Pathguide [5], (e.g. DrugBank [6], STITCH [7], CTT [8], CTD [9] and BindingDB [10], et al), a disease-specific searching platform is still needed to fully understand drug effects on the human body.

A new cancer systems biology approach to drug discovery has emerged in recent years. The primary focus of this paradigm is to understand the actions of drugs by considering targets in the context of the biological networks. By focusing on a systems level, it provides a better way to examine complicated diseases that can be caused by several gene mutations, such as cancer [11]. However, most methods published so far focus on modeling the structure of the drug target network qualitatively [12]. To examine a drug's effect on a molecular network representative of the disease, more quantitative and accurate modeling techniques need to be developed by utilizing the concept of network pharmacology [11] or network medicine [13].

In post-genome biology, molecular connectivity maps have been proposed to establish comprehensive knowledge links between molecules of interest in a given biological context [14]. Molecular connectivity maps between drugs and genes/proteins in a disease-specific context can be particularly valuable because they allow researchers to evaluate drugs against each other using their unique gene/protein-drug association profiles. The functional approach to drug comparisons helps researchers gain global perspectives on both the toxicological profiles and therapeutic profiles of candidate drugs. Furthermore, the time it takes to develop high quality drugs in new therapeutic areas can also be reduced by using this method.

One approach for developing molecular connectivity map data is to generate disease-specific protein-drug association profiles computationally by mining bio molecular interaction networks and PubMed literature [15]. The Connectivity Maps (C^2^Maps) web server [16] is an online bioinformatics resource that provides biologists with potential relationships between drugs and genes/proteins in specific disease contexts based on network mining and literature mining. It's based on the concept of network pharmacology by examining many drugs at the same time and studying the drug disease relationship based on the underlying protein interaction network instead of drugs' direct target. C^2^Maps provides quantitative measurements of protein's and drug's relevance to a specific disease by applying networking mining and the statistical testing methods in text mining and thus offers new insight to assess overall drug efficacy and toxicity.

Occurrences between proteins and drugs from literature mining of C^2^Maps don't necessarily tell research what type of relationships they have, therapeutic or toxic. To overcome these limitations, we further standardize the classifications between proteins and drugs and then perform literature curations to determine drugs' effect on proteins on higher resolutions. Such valuable information is not readily available from the existing drug-target (protein-compound interaction) databases (e.g. DrugBank, STITCH, CTT, et al) though they may be scattered within a description or referenced text.

To assess drug pharmacological effect, such as drug efficacy and toxicity, we assume that "ideal" drugs for a patient diagnosed with a certain disease should modulate the gene expression profiles of this patient to the similar level with those in normal healthy people. Therefore, for those statistically over-expressed genes, drugs should be able to inhibit their expression level to the normal range. Similarly, for those statistically under-expressed genes, drugs should be able to activate their expression level to the normal range. In this way, drugs can treat or prevent the disease through reversing the gene expression level from disease status to the normal range, thus modulating cellular function as in normal cells.

By assuming that if the gene expression profiles of disease and drug are opposing, then the drug might be a potential treatment option of the disease, [14] identified novel drug indications in diet-induced obesity or Alzheimer's disease. Another work by Atul [17] utilized the same gene expression data and algorithms with large scale gene expression data from GEO to study associations between 100 diseases and 164 drug molecules. They found candidate therapeutics for 53 of the diseases. These studies are proof of principle that how using public genomics database and similar hypothesis can benefit drug discovery. Though gene expression data are publicly available for more than 1000 compounds in the second release of [14], yet there are numerous compounds that are not part of the database. Another limitation of this overly simplified hypothesis lies in it doesn't differentiate important genes from unimportant ones. Ideally a biological meaningful scoring methods needs developed.

Drugs effect data from literatures could be complementary here. In this work, we focus on building comprehensive disease-gene-drug connectivity relationships with drug-protein directionality (inhibit/activate) information based on the C^2^Maps platform [16]. To show the feasibility of applying the data for computational drug discovery, we advanced previous hypothesis forward by assigning different weights to different genes. However this work aims to provide the data for the future network pharmacology research instead of developing a drug efficacy prediction method .This work has the following contributions:

1) It's the first time that we have published this comprehensive C^2^Maps database server. Although [16] provides the underlying computational methodology, it only covers a small number of diseases such as Alzheimer's Disease.

2) We create an interactive interface for directionality annotation of drug-protein pairs with literature evidences from PubMed.

3) We curate the directionality information of drug protein pairs for three disease phenotypes: breast cancer, colorectal cancer and Alzheimer disease from 5133, 4869 and 3928 PubMed abstracts, respectively. We also upload these curated directionality information into the C^2^Maps, and perform a statistical analysis on them. Curation of additional diseases, like pancreatic cancer and autism, is still on-going.

4) We enhance the functionality of disease-specific searching for relevant proteins and drugs with directionality information.

5) We update the comprehensive disease-gene-drug connectivity data in the C^2^Maps databases, including 19,569,563 PubMed abstracts in the current version and 142,523 unique 3 star protein interactions in the current version.

6) We also use breast cancer as a case study to demonstrate the functionality of disease-specific searching for relevant drug-protein pairs with directionality information.

7) Based on the searching result, we show the feasibility of performing drug pharmacological effect evaluation for two important breast cancer drugs to show the power of updated C^2^Maps in drug efficacy and toxicity assessment.

## Materials and methods

### Data sources and systems design

**Figure 1:**
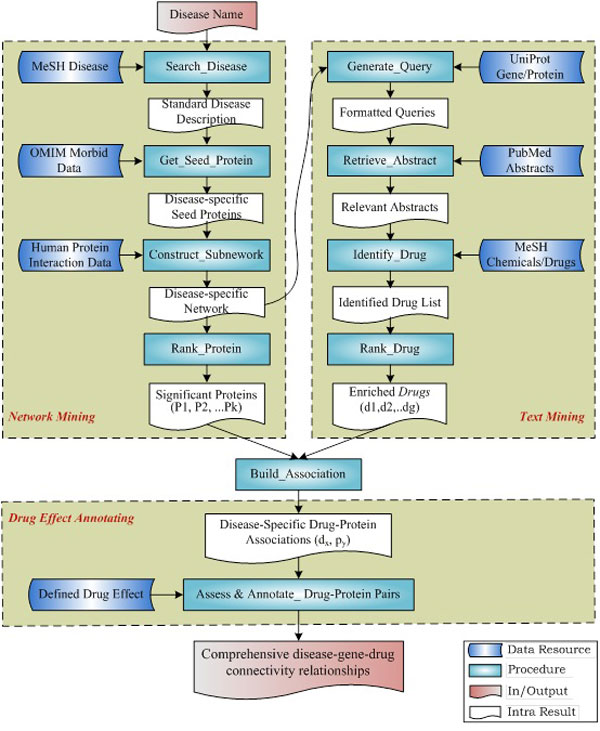
**CMap workflow for a given disease-specific study**.

As shown in Figure 1, the C^2^Maps platform incorporates three major components in its systems design:

• *Network mining *component takes a query disease term as the input, and generates a ranked list of disease-relevant proteins as the output, through 1) MeSH term matching, 2) disease-associated gene searching from OMIM [18], 3) network expanding in HAPPI [19], and 4) network-based protein ranking;

• *Text mining *component takes an input list of genes or proteins, and creates a list of enriched disease candidate drugs that are significantly associated with the disease-relevant proteins from the previous component as the output, through 1) gene/protein name mapping using UniProtKB, 2) article abstract retrieving from PubMed, 3) drug/chemical compound identification using MeSH term, and 4) disease-specific drug-protein pair ranking;

• *Drug effect annotating *component can allow users to 1) retrieve disease-specific drug-protein association list, 2) curate drug-protein directionality information from PubMed abstract, 3) annotate these drug-protein directionality information interactively, and 4) browse disease-specific drug-protein directionality information online.

In specific, we apply the network mining method originally developed by Chen et al. [20] to fish cancer relevant proteins from the protein interaction network. We expand cancer related genes/proteins using PPIs recorded in the Human Annotated and Predicted Protein Interactions (HAPPI) database to construct cancer-specific PPI sub-network. A protein's cancer relevance score *r_p _*is calculated as the function (1).

(1) $$r_{p}=k\text{ln}\left(\sum_{q\in NET}conf\left(p,g\right)\right)-\text{ln}\left(\sum_{q\in NET}N\left(p,q\right)\right)$$

Here*, p *and *q *are indices for proteins in the cancer-related interaction network *PPI*, *k *is an empirical constant (*k*=2 in this study), *conf(p, q) *is the confidence score assigned to each interaction between protein *p *and *q*, and *N(p, q) *holds the value of 1 if the protein *p *interacts with *q*.

To retrieve the important drugs for cancers and to parse out drug terms in the articles, we acquire PubMed abstracts with the list of cancer-related genes/proteins derived earlier from PPI as queries. Drug term frequency is calculated and compared to term statistical distributions from the entire PubMed abstracts to get the p-value of drugs using function (2).

(2) $$\Delta_{j}=\frac{\left(\bar{df\left(d_{j}|{T^{\prime }}_{NET}\right)}-\bar{df\left(d_{j}|{T^{\prime }}_{Random}\right)}\right)}{\sqrt{\frac{Var\left(d_{j}|{T^{\prime }}_{NET}\right)}{N_{NET}}+\frac{Var\left(d_{j}|{T^{\prime }}_{Random}\right)}{N_{Random}}}}$$

Here, $T_{NET}^{{}^{\prime }}=\left\{T_{NET1}^{{}^{\prime }},T_{NET2}^{{}^{\prime }},...\right\}$ is generated by sampling the entire collection of retrieved abstracts $T_{NET}^{{}^{\prime }}$. $N_{NET}^{}=\left|T_{NET}^{{}^{\prime }}\right|$ is the size of each sample. $T_{Random}^{{}^{\prime }}=\left\{T_{Random1}^{{}^{\prime }},T_{Random2}^{{}^{\prime }}...\right\}$ refers to a random sample generated by randomly sampling the entire number of PubMed abstracts; the size of the random sample is $N_{Random}$. $df\left(d_{j}|T_{NET}^{{}^{\prime }}\right)$ and $df\left(d_{j}|T_{Random}^{{}^{\prime }}\right)$ refer to average document frequencies of $d_{j}$ in $T_{NET}^{{}^{\prime }}$ and $T_{Random}^{{}^{\prime }}$. $Var\left(d_{j}|T_{NET}^{{}^{\prime }}\right)$ and $Var\left(d_{j}|T_{Random}^{{}^{\prime }}\right)$ refer to document frequency variances of $d_{j}$ in $T_{NET}^{{}^{\prime }}$ and in $T_{Random}^{{}^{\prime }}$. A two-sided tails t-test was then performed to calculate the p-value. A thorough description of the computational components and algorithms used, along with data sets and data processing parameters, is described in detail by Li et al. [16].

The C^2^Maps platform follows a multi-tier architecture design. The back end was implemented as PL/SQL packages in the Oracle 11 g database server, with the Oracle Text engine enabled, to ensure scalable querying of PubMed text documents. The C^2^Maps application middleware was implemented in the Oracle Application Express (APEX) server, which bridged between the Apache web server and the Oracle database server.

The current release of C^2^Maps uses the following data sets: 19,569,563 records in the PubMed/MEDLINE baseline database [21], 142,523 human protein-protein interactions above 3-star confidence ratings in the HAPPI database [19], 26,142 descriptors in the MeSH database (Category C for diseases and Category D for chemicals and drugs) [22], 20,331 entries for the curated human proteins in the UniProtKB database [23], 18,344 entities in the OMIM database [24], and 4,772 entities in the DrugBank. Current statistics for the included database records is also shown in Table 1. We manually curated the top 500 drug-protein pairs for 'Alzheimer disease', 'Breast cancer' and 'Colorectal cancer' from C^2^Maps by assigning the effects of drugs on proteins as defined in the next section. As a result, C^2^Maps platform contains 3928, 5133 and 4869 curated records for Alzheimer disease, Breast cancer and colorectal cancer, respectively. All data is warehoused in a local Oracle 11 g database.

**Table 1:** Current statistics for the included database records

Dataset	Data Resource	Record count
**Biomedical Literature**	PubMed	19,569,563
**Human Protein-Protein Interaction**	Unique HAPPI 3-star interactions	142,523

**Disease and Drug Terminology**	MeSH descriptors	26,142
**Human Protein**	UniProtKB	20,331

**Disease-Gene relationships**	OMIM	18,344
**Drug Information**	DrugBank	4,772

### Drug effect annotation

**Figure 2:**
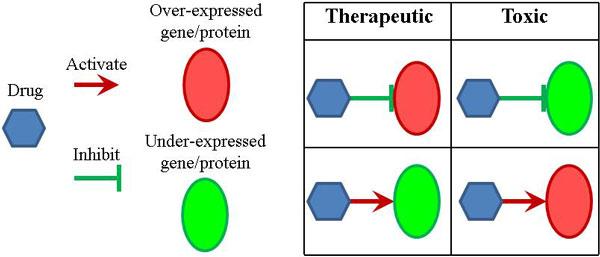
**Illustration of drug pharmacological effects based on directionality information for drug-protein pairs**.

Since our hypothesis is that ideal drugs for a patient diagnosed with a certain disease should modulate the gene expression profile of this patient to the similar level with those in healthy people, we annotate a drug's pharmacological effect on a protein using one of the following three categories (also illustrated in Figure 2):

• *Therapeutic*: if the drug activates the under-expressed protein or inhibits the over-expressed protein, we define that the drug has a therapeutic effect on that protein

• *Toxic*: if the drug activates the over-expressed protein or inhibits the under-expressed protein, we define that the drug has a toxic effect on that protein

• *Ambiguous*: if there is missing directionality information for either the nodes (i.e. proteins/drugs) or edges.

### Perturbation effects of drugs on proteins/genes

We use breast cancer as an example to illustrate how we curate the directionality of a drug-protein pair retrieved from the C^2^Maps platform. The following are four categories of an annotated drug-protein relationship pair (also, refer to Table 2):

**Table 2:** Curation of drug-protein relations from Pub-Med abstracts

Relation	Protein	Drug	PMID	Relation Proof
**Up-Regulated**	BRCA1	Estradiol	7553629	"BRCA1 mRNA and protein levels were significantly decreased in estrogen-depleted MCF-7 and BT20T cells and **increased again **after stimulation with beta-estradiol".
**Down-Regulated**	P53	Cycloheximide	9484835	"Treatment of cells with cycloheximide (CHX) **prevented the activation **of p53 in all phases of the cell cycle and its accumulation in G1/S and S".

**Indirect**	BRCA1	Hydroxyurea	9267023	"Hydroxyurea-mediated DNA synthesis **arrest of S phase MCF7 cells led to a loss of BRCA1 **from these structures".
**Ambiguous**	GCR	Dexamethasone	12974633	"GRalpha and GRbeta transcripts are coordinately **upregulated**in CEM-C7 cells and coordinately **downregulated **in IM-9 cells by dexamethasone".

**Unknown**	AKT1	Phosphothreonine	11087733	The drug-protein relation is not mentioned in the text.

• *Activation *- "Subsequent injection of tamoxifen triggers the transient activation of Akt/PKB in mice." (Tamoxifen and AKT1_HUMAN, PMID: 12640620).

• *Inhibition *- "Treatment of cells with Cycloheximide (CHX) prevented the activation of p53 in all phases of the cell cycle and its accumulation in G1/S and S." (P53_HUMAN and Cycloheximide, PMID:9484835).

• *Indirect Yes *- "Hydroxyurea-mediated DNA synthesis arrest of S phase MCF7 cells led to a loss of BRCA1 from these structures." (BRCA1_HUMAN and Hydroxyurea, PMID:9267023).

• *Ambiguous *- "GRalpha and GRbeta transcripts are coordinately upregulated in CEM-C7 cells and coordinately downregulated in IM-9 cells by dexamethasone." (GCR_HUMAN and Dexamtheasone, PMID:12974663).

We can see that the literature does contain such information and thus provides basis for the directionality information retrieval. We focus on curating drug actions in the disease context rather than only in cell lines like in [14]. Different research works under specific contexts may produce conflicting conclusions regarding drug protein relationship. Take Tamoxifen and estrogen receptor as examples. As shown in Table 3, we successfully extracted 7 article abstracts which support the inhibitory effects of Tamoxifen on estrogen receptor and 2 PubMed abstracts which support the stimulatory effects of Tamoxifen on estrogen receptor. The pre-dominant evidence showing Tamoxifen's inhibition on estrogen receptor [25] matches well with the fact that Tamoxifen acts as an antagonist for estrogen receptor. Beside checking the majority vote of all the related papers, we also traced back the original references. For Tamoxifen, it inhibits estrogen receptor in the mammary tissue while activating estrogen receptor in bone density. In our breast cancer case study, we decided Tamoxifen inhibits estrogen receptor because the gene expression experiment was based on breast tissue. In the future, we plan to add additional contexts such as experimental conditions, disease subtypes and so on. In the current version, they are not added due to limited availability of those data in abstracts.

**Table 3:** PubMed evidence for Tamoxifen's effect on ESR1

Drug	Protein	PMID	Direction
Tamoxifen	ESR1_HUMAN	14507640	-1
**Tamoxifen**	ESR1_HUMAN	2359140	-1
**Tamoxifen**	ESR1_HUMAN	14759988	-1

**Tamoxifen**	ESR1_HUMAN	11774281	-1
**Tamoxifen**	ESR1_HUMAN	2137212	-1

**Tamoxifen**	ESR1_HUMAN	9328205	-1
**Tamoxifen**	ESR1_HUMAN	11261829	-1

**Tamoxifen**	ESR1_HUMAN	12767276	1
**Tamoxifen**	ESR1_HUMAN	11812086	1

1 represents that the drug up regulates the protein while -1 represents down regulation

### Data access and website usage

**Figure 3:**
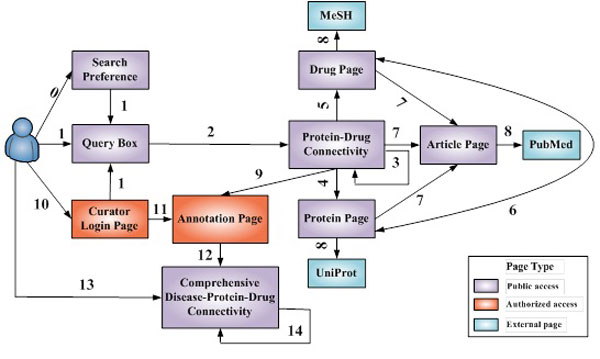
**The navigational site map of the CMap platform**. The numbers refer to: 0. Configure search preference; 1. Input interest disease; 2. Generate Protein-Drug connectivity; 3. Refine initially constructed connectivity map; 4. Link to protein page; 5. Link to drug page; 6. Link between protein and drug pages; 7. Link to evidence article pages; 8. Link to external data resources (MeSH, UniProt and PubMed); 9. Import enriched disease specific protein-drug associations for further annotation; 10; Authorized users (curators) set up profiles and login in; 11. Annotate effects of drugs on protein/genes; 12. Release annotation results; 13. Browse annotated disease-protein-drug connectivity relationships; 14. Filter or search for interested subset of connectivity relationships.

**Figure 4:**
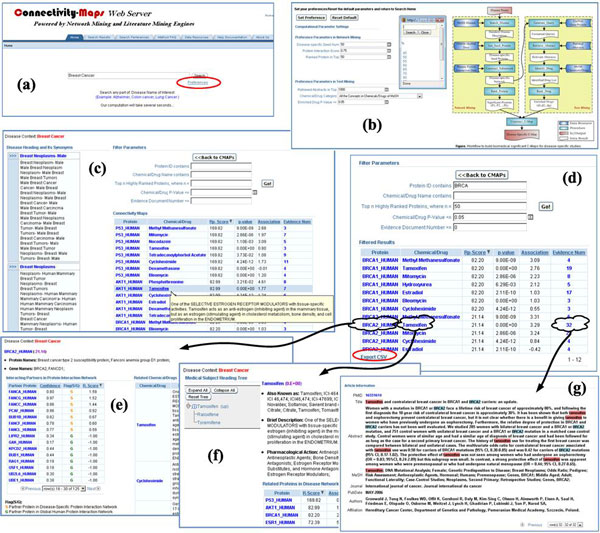
**Web Interface for C^2^Maps basic query function**. (a) C**^2^**Maps default home page with a query box; (b) Preference configuration page with editable parameters in network mining component and text mining component; (c) Disease-specific protein-drug connectivity map page shows disease-relevant proteins, their ranking score, and drugs, their p-value, their relative frequency, as well as evidences supporting the protein-drug associations; (d) Filter result page shows the subset of the connectivity map; (e) Protein detail page shows the disease-related protein, its partner proteins in interaction network, and related drugs in retrieved abstracts; (f) Drug detail page shows the disease-related drugs, its neighbor drugs in Medical Subject Heading Tree, and related proteins in disease-specific protein interaction network; (g) Article detail page shows the literature references in PubMed with protein entity and drug entity highlighted.

The C^2^Maps online platform (http://bio.informatics.iupui.edu/cmaps) provides researchers a web-based bioinformatics user interface, following principles described in [26]. As shown in Figure 3, users can begin with a single disease term as a query and navigate to extract significant subsets of the disease-specific C^2^Maps. We also show snapshots of C^2^Maps output web pages in Figure 4, using "Breast Cancer" as a query example. The statistically significant relationships between proteins and drugs that are extracted from the literature are then displayed in tabular format (Figure 4). The proteins, drugs, and evidence numbers are further linked to protein, drug, and the article detail page, respectively. The search results can also be sorted by the protein ranking score (R-Score) and Chemical/Drug significance (P-Value). In addition, the page also lists Disease Context (disease name of user interest) and Disease Terminology (disease name containing query term in the controlled vocabulary of Medical Subject Headings). Users can also specify advanced search criteria for further biological/pharmacological analysis. Annotations for the extracted relations could be performed from the 'Annotate Data' tab.

### Browsing disease-specific drug-protein relationship information

**Figure 5:**
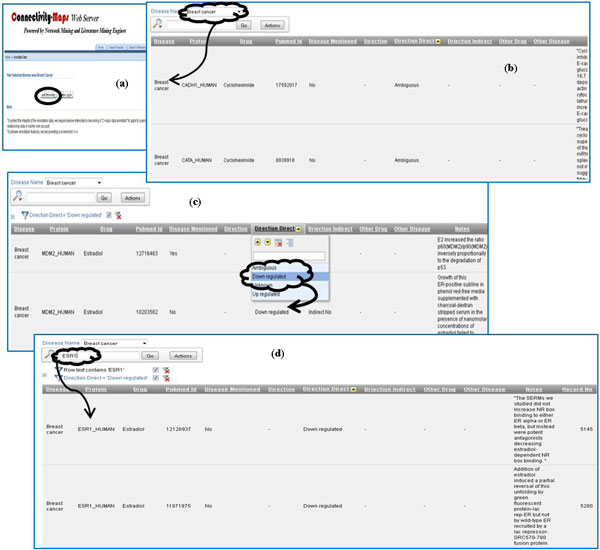
**Web Interface for CMap Annotation data browse**. (a) Annotation Data: main search page allowing either public browse or authorized curation; (b) Annotation Data Browse: displaying curated directionality between drug and proteins for certain disease; (c) Filtering: Each column support filtering (e.g., only show 'Down regulated' directionality); (d). Search function: search by either drug or protein. For example, search all records for ESR1 protein.

Any public C^2^Maps database user can access the well curated drug-protein directionality data. The database will display disease, protein, drug directionality, and PudMed evidence for each record. Each column can be sorted or filtered. One can also display only drug-protein directionality belonging to certain disease by selecting it from the drop down list (Figure 5b). Furthermore, the user can also search the keywords, such as protein name or drug name, to retrieve only specific records. Currently, the C^2^Maps platform contains 3928 curated records for Alzheimer disease, 5133 curated records for breast cancer, and 4869 curated records for colorectal cancer. More curation information will be updated regularly.

### Interactive interface for directionality annotation

**Figure 6:**
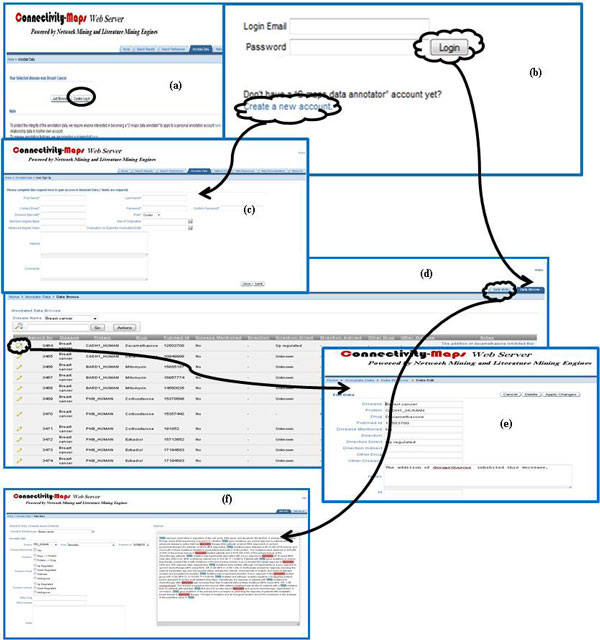
**Web Interface for CMap Annotation data curation**. (a) Annotation Data: main search page allowing either public browse or authorized curation; (b) Curator login: require login to curate directionality for certain disease; (c) User sign up: application form to create an account as a curator; (d) Data Browse: the browse page after login; (e) Data Edit: update or delete previously curated records; (f) Data Entry: 1. select disease; 2. relevant protein based on 1; 3. relevant drug based on 2; 4. relevant PubMed ID based on both 2 and 3; 5. relevant abstract based on PubMed ID from 4; 6. Curate directionality based on 5.

An authorized C^2^Maps database user can also annotate selected C^2^Maps contents by performing manual curation from the 'Annotate Data' tab. The user may apply for an annotator's account to edit protein-drug interactions suggested by the C^2^Maps automated recommendation system. This editing is provided through a separate user interface that enables the annotator to categorize protein-drug relationships as direct (including activation, inhibitory, ambiguous), indirect, or unknown (Figure 6f). The user will first select the assigned disease and the C^2^Maps webserver will populate disease relevant protein and drug pairs. All the PudMed abstracts mentioning both the relevant protein and drug will be pulled out and the curator can read the abstract to annotate the directionality between the drug and the protein. The user can also edit (Figure 6d) each record or delete it.

## Results

### Statistical analysis for reliability

The major computational components of the C^2^Maps platform were developed using validated computational techniques. In the network mining component, protein interaction network expansion was able to reduce the initial biases and low data coverage, which may have existed in the seed list of protein. We used the new HAPPI database instead of other protein interaction databases because of its overall better data quality (comparable or better than data in the HPRD database for quality star grades of 3 and above) and coverage (more than 280,000 human protein interactions with star grades of 3 and above), which was thoroughly described in Chen et al. [19]. In the text mining component, the PubMed abstract retrieval for each protein was shown to improve Information Retrieval (IR) recall performance without sacrificing precision The quality of disease drug identifications was shown to outperform comparable systems with balanced sensitivity, specificity, and positive predictive values (for details, refer to Li et al. [16]).

In Table 4, we show a summary of C^2^Maps platform performance, by comparing its overall sensitivity, specificity, PPV (positive predictive value), F-score, and ACC (accuracy) measures among a number of cancers. The result confirmed that C^2^Maps performed well consistently across different disease studies.

**Table 4:** Performance assessment of C^2^Maps in varying cancers.

	Bladder	Breast	Leukemia	Lung	Lymphoma	Melanoma	Ovary	Pancreas	Prostate
Sensitivity	80.84%	79.80%	83.16%	78.44%	81.20%	77.39%	80.88%	84.99%	82.84%
**Specificity**	87.11%	84.91%	86.11%	89.37%	87.60%	91.53%	84.34%	86.45%	88.38%
**ACC**	86.70%	84.27%	85.63%	87.86%	86.78%	90.17%	84.09%	86.34%	87.93%

**PPV**	30.51%	43.01%	53.39%	54.06%	48.92%	49.24%	28.84%	33.82%	38.88%
**F-Score**	44.30%	55.89%	65.03%	64.01%	61.05%	60.19%	42.52%	48.38%	52.92%

The experiment were performed using a protein interaction confidence minimum threshold of 3 star and above (i.e., reliability score of >0.75) and retrieved drug p-value at a minimum threshold of 0.05. The detailed evaluation procedures and measurement definitions, they can be found in the "Method FAQ" page of our C^2^Maps website and as supplemental materials.

### A case study on breast cancer specific searching for relevant drug-protein pairs with directionality information

**Figure 7:**
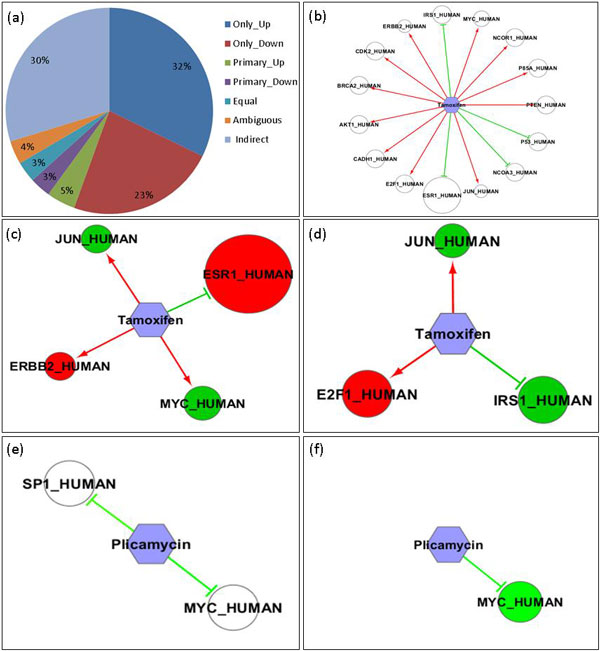
**Breast cancer case study for drug pharmacological effect evaluation with C^2^Maps**. (a) Distribution of directionality categories for breast cancer; (b) A drug-target subnetwork with directionality information specific for Tamoxifen; (c) Drug effect evaluation for Tamoxifenon breast cancer subtype - luminal A; (d) Drug effect evaluation for Tamoxifenon breast cancer subtype - basal-like; (e) A drug-target subnetworkwith the directionality information specific for Plicamycin; (f) Drug effect evaluation for Plicamycinon breast cancer subtype - luminal A.

We evaluated breast cancer drugs from C^2^Maps based on our hypothesis. First, we obtained top 500 drug protein pairs for breast cancer from the C^2^Maps web server, 23 drugs and 103 proteins, respectively. The *r_p _*scores for those proteins range from 1.69 to 169.82 and the P-Values for those drugs are all below 0.05. Well known breast cancer related proteins, like BRCA1, or related drugs, like tamoxifen, were included in these 500 pairs. We then examined all supporting evidence for each drug protein relations, a total of 5,225 PubMed abstracts, and manually curated them to extract the relevant drug effect information. Out of those 500 pairs, 155 pairs contained information of how the drug affects the protein in the literature, yielding a total of 19 drugs and 52 proteins. After performing manual curation, 79 drug protein pairs contained only up-regulation information, 57 only down-regulation information, 11 primarily up-regulation information, and 8 primarily down-regulation information. The distribution of directionality categories for breast cancer is shown in Figure 7a.A subnetwork based on the directionality information specific for Tamoxifen can be constructed from C^2^Maps directionality data (shown in Figure 7b). Another subnetwork based on the directionality information specific for Plicamycin is also shown in Figure 7e.

### A case study on drug efficacy evaluation with C^2^Maps

Drug efficacy can be measured by the ability of a drug to produce the desired phenotypic effect or molecular effect. To evaluate drug efficacy in the molecular level based on our hypothesis illustrated in Figure 2, we need to know how drugs can affect the expression of its interacting genes and how those genes are expressed in disease conditions. We have got the former from the above case study of C^2^Maps. To get the latter, we performed differential analysis on a well-studied microarray dataset-GSE3191[27]. This experiment contains breast cancer subtype luminal A, basal-like and also normal breast tissues. We obtained the differential genes for both breast cancer subtypes - luminal A and basal-like when compared to normal. We identified 579 differential genes between luminal A and normal, 773 differential genes between basal like and normal. We used these two sets for the following case study.

### Tamoxifen efficacy and toxicity assessment for the breast cancer subtype - luminal A

Tamoxifen is a standard drug clinically used for breast cancer and has 15 interacting proteins with directionality annotations from C^2^Maps (shown in Table 5).We intersected differential genes from luminal A microarray experiment with Tamoxifen's interacting partners from C^2^Maps. Four proteins out of 15 are differentially expressed between luminal A and normal, including ESR1. In Figure 7c, drugs are represented as hexagons and proteins as circles. The size of a protein node is proportional to the *r_p _*score, an indication of importance of this protein to breast cancer. Red nodes stand for over-expressed proteins in breast cancer while green ones represent under-expressed proteins. For edges between drugs and proteins, red symbolizes that the drug activates the protein while green symbolizes inhibition. From the Figure, Tamoxifen has 3 therapeutic effects: it inhibits over-expressed ESR1, activates under-expressed JUN and activates MYC. Tamoxifen also has one toxic effect, activating over-expressed ERBB2, which might help explain certain side effects when using Tamoxifen. Considering that ESR1 is more significant for breast cancer compared with the other three proteins, overall, Tamoxifen has more of a therapeutic value in Luminal A patients by reversing the gene expression of important disease proteins in the network level (Figure 7c).

**Table 5:** Tamoxifen relevant proteins and their directionality

Drug	Protein	RpScore	Association	Direction
Tamoxifen	AKT1_HUMAN	82.99	1.77	1
Tamoxifen	BRCA2_HUMAN	21.14	3.29	1
Tamoxifen	CADH1_HUMAN	17.57	0.95	1
Tamoxifen	CDK2_HUMAN	2.94	2.12	1
Tamoxifen	E2F1_HUMAN	2.95	1.59	1
Tamoxifen	ERBB2_HUMAN	2.07	3.19	1
Tamoxifen	ESR1_HUMAN	72.39	5.11	-1
Tamoxifen	IRS1_HUMAN	2.51	1.29	-1
Tamoxifen	JUN_HUMAN	2.91	1.51	1
Tamoxifen	MYC_HUMAN	3.49	2.5	1
Tamoxifen	NCOA3_HUMAN	2.61	3.01	-1
Tamoxifen	NCOR1_HUMAN	2.81	4.05	1
Tamoxifen	P53_HUMAN	169.82	0.8	-1
Tamoxifen	P85A_HUMAN	2.92	1.57	1
Tamoxifen	PTEN_HUMAN	3.98	0.98	1
Plicamycin	MYC_HUMAN	3.49	4.16	-1
Plicamycin	SP1_HUMAN	3.32	6.24	-1

1 represents that the drug up regulates the protein while -1 represents down regulation

### Tamoxifen efficacy and toxicity assessment for the breast cancer subtype - basal-like

In Figure 7d, we portray the drug protein interaction for Tamoxifen in basal patients. Three proteins out of its 15 interacting proteins are differentially expressed between basal patients and normal. Tamoxifen has only 1 therapeutic effect by activating under-expressed JUN, while 2 toxic effects by activating over-expressed E2F1 and inhibiting under-expressed IRS1. However, all these three proteins are relatively insignificant for breast cancer. This implies a neutral role overall when using Tamoxifen in basal patients since it is not able to reverse its interacting proteins in basal condition (Figure 7d). This agrees well with the clinical fact that basal or triple negative breast cancer patients fail to benefit from Tamoxifen treatment.

### Plicamycinefficacy and toxicity assessment for the breast cancer subtype - luminal A

Plicamycin was an approved antineoplastic antibiotic for a variety of advanced forms of cancer. It has been withdrawn from market in 2000. In Figure 7f, we showed the drug protein interaction for Plicamycin in Luminal A patients. It has 2 interacting proteins with directionality annotations (shown in Table 5) and both are not significant in breast cancer with a low *r_p _*score. Only 1 protein out of these 2 is differentially expressed between luminal A and normal. Plicamycin has a toxic effect overall by inhibiting under-expressed MYC. This implied a neutral or toxic effect when using Plicamycin in Luminal A subtype breast cancer patientssince it is not able to reverse its interacting proteins in the disease condition (Figure 7f). This may help explain why it was withdrawn in 2000.

## Conclusions

In this study, we present an upgraded C^2^Maps platform to evaluate drug pharmacological effects based on the hypothesis that an ideal drug can reverse the gene expression level in a disease back to those in normal conditions. This online platform will enable users to query high-coverage protein-drug connectivity maps in real time. It enables users to research up-to-date knowledge of connectivity maps for a specific disease, explore therapeutic protein targets, design repurposed drug compounds, and assess toxicological impacts of drug compounds on disease-relevant genes/proteins. Three efficacy case studies prove the feasibility to apply the literature mined drug directionality data from C^2^Maps for drug efficacy study. It will be a major resource to biomedical researchers interested in developing disease-specific therapeutic and diagnostic applications based on progresses in network biology and network pharmacology.

## Discussion

From the case study on breast cancer drug effect evaluation, we can see that there is still room for improvement, although the two breast cancer drugs were well-evaluated. The information for judging whether a drug has global therapeutic effects on other diseases is limited due to the manual curation procedure. This information is very valuable for drug repurposing, however. In the current version of C^2^Maps, drug-protein pairs are mainly come from literature mining based on disease-gene searching and network mining, which can be supplemented by plenty of publicly-available drug target databases. We will continue to update C^2^Maps and improve its usability through achieving the following functionalities in the near future.

1) We will increase the functionality of drug-orientated searching for relevant disease phenotypes and proteins in the C^2^Maps. It will allow users to input drug names, not just disease names. It should be able to retrieve all the disease names and genes/proteins related to this drug. This function will be very useful for drug repurposing.

2) We will increase the functionality of disease-orientated browsing for relevant proteins and drugs in the C^2^Maps by using disease phenotype trees. It will allow users to browse the database by clicking the disease name. The current version only supports the disease-specific searching function without any browsing function.

3) We will also enhance the functionality of interactive directionality information annotation for drug-protein pairs in the C^2^Maps by using natural language processing (NLP) techniques. The literature curations for breast cancer, colorectal cancer and Alzheimer disease took three experts nearly one year's effort to complete. While this ensures the data quality, it's time consuming. With those golden standard dataset from curation, we will NLP techniques to allow users to curate and annotate directionality information from PubMed abstracts more easily and semi-automatically.

## Competing interests

The authors declare that they have no competing interests.

## Authors' contributions

JYC conceived this work, guided the research team by providing ideas and feedback along the way, and revised the manuscript. HH involved the drug efficacy evaluation hypothesis development, carried out annotation web page development, designed the case studies and wrote the manuscript. XW participated in the drug efficacy hypothesis initiation, directionality concept development, case studies and manuscript writing. RP updated the PubMed database and tables in the Apex application. JL developed the previous version of C^2^Maps and performed case studies about the web server in her paper. GZ helped with annotation web page development.SI performed the directionality curations for breast cancer and colorectal cancer. All authors read and approved the final manuscript.
